# Characterization and *in vitro* antibacterial activity of sulfated polysaccharides from freshwater alga *Cladophora crispata*

**DOI:** 10.1099/acmi.0.000537.v5

**Published:** 2023-07-26

**Authors:** Mohanad Khaled Jasem, Abd-Alwahab Merai, Adnan Ali Nizam

**Affiliations:** ^1^​ Food Sciences Department, Faculty of Agriculture, Damascus University, Damascus, Syria; ^2^​ Plant Biology Department, Faculty of Science, Damascus University, Damascus, Syria

**Keywords:** antibacterial activity, *Cladophora crispata*, functional groups, minimum inhibitory concentration (MIC), quantity and quality determinations, sulfated polysaccharides

## Abstract

Barada River is characterized by an abundant growth of freshwater algae. *Cladophora* sp. algae have emerged as a new source of bioactive compounds. In this research *Cladophora crispata* was cultivated with the outdoor method, and algal sulfated polysaccharides (SPs) were extracted by an ultrasonic-assisted extraction method. After extraction, gel filtration was used to purify the crude SPs, SP compounds were determined and selected, and the effect of purified SPs as antibacterial agents was investigated. The purified extract gave two fractions (F1 and F2). The chemical components of both crude and purified SPs were then determined. The highest carbohydrate content (74.12%) and protein content (4.02%) was found in the crude extract, while the highest sulfate content (12.17%) was found in purified fraction F2, and the highest uronic acid content (18.46%) was found in purified fraction F1. Fourier transform infrared spectroscopy (FT-IR) was used to confirm that the crude extract and fractions consist of sugar, uronic acids, protein and sulfate groups. Both F1 and F2 consisted of rhamnose, galactose, xylose and ribose based on high performance liquid chromatography (HPLC) separation. Each fraction showed an inhibitory effect on Gram-positive and Gram-negative bacteria. F2 has the lowest minimum inhibitory concentration (MIC) value against *Staphylococcus aureus *, *Bacillus anthracis *, *Enterobacter aerogenes* and *Pseudomonas aeruginosa *, where its MIC values were 6, 13, 25 and 30 mg ml^−1^, respectively. Algae polysaccharides are of key interest due to their antibacterial properties, which has led to them being included in pharmaceutics and food applications.

## Graphical Abstract

Sulfated polysaccharides (SPs) from Cladophora crispata were extracted by ultrasonic bath: purify the crude SPs. Compounds of SPs were identified, and we investigated the effect of SPs as antibacterial agents.
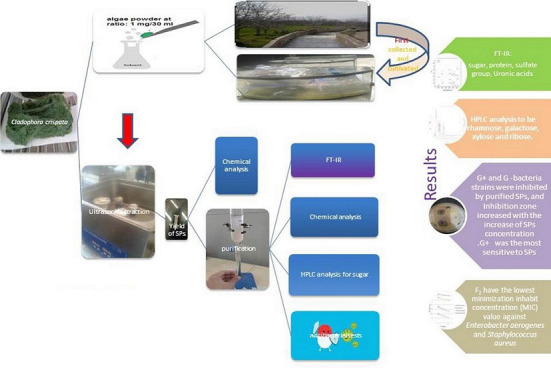

## Highlights

- Sulfated polysaccharides (SPs) were extracted from *Cladophora crispata* using an ultrasonic bath.
- SPs consist of sugar, uronic acids, protein and sulfate groups.
- Gram-positive bacteria are more sensitive to SPs.

## Data Summary

All supporting data, code and protocols have been provided within the article.

## Introduction

Green algae (Chlorophyta) are the primary producers in aquatic ecosystems, with an estimated count of 6000 to 8000 species. Most of these are macroalgae, while others are microalgae such as *Cladophora* (Ulvophyceae, Cladophorales) [1]. These are multinucleate filamentous algae, and their cells contain a parietal perforate or reticulated chloroplast. The presence of pigments such as chlorophyll (a and b), xanthophylls and β-carotene give it its bright green colour. *Cladophora* is predominantly benthic [2], and it is usually found in the region of unidirectional flow or periodic wave action in fresh water and marine habitats. *Cladophora* is a mid to late successional species [1, 2].

Algae can be cultivated in open and closed ponds, and photobioreactors for biomass production [3]. Microbial polysaccharides are of great biotechnological and commercial interest, and have a wide application in the food, cosmetics and medical industries due to their emulsifying, thickening, flocculating, stabilizing, anti-oxidizing and antimicrobial properties. It is much easier and advantageous to use them because of the short life cycle of the microbes that allows quick production under controlled environmental conditions. Polysaccharides have been extracted from fungi, bacteria and yeasts [4–6], and they are the most important products of algae, representing 38 to 54 % of algal dry weight [7, 8]. They show important biochemical properties, which require further investigation to understand their structure and biochemical functions. Several studies have reported the antibacterial, antifungal and antiviral properties of sulfated polysaccharides (SPs) [9–11], as well as their scavenging activity against superoxide, hydroxyl, DPPH (1,2-diphenylpicrylhydrazyl) and ABTS radicals [2,2′-azino-bis (3-ethylbenzothiazoline-6-sulfonicacid) diammonium salt] [12, 13], and they have commercial applications in food industries [14]. Moreover, the SPs from *Cladophora oligoclada* have been reported to have anticoagulant properties [15]. Polysaccharides from green algae are heteropolysaccharides that are composed of different monosaccharides [10, 16].

Algae polysaccharides can be extracted by several green techniques such as microwave, enzyme and ultrasonic-assisted extraction (UAE). UAE reduces energy consumption and solvent use. In addition, the low temperatures and short times used in UAE processes can maintain the functionalities of the bioactive compounds [12, 17, 18]. The objectives for this study were (a) to isolate and cultivate *Cladophora crispata* to understand its growth dynamics, and (b) to study the chemical and functional properties of the crude and purified SPs produced by this species.

## Methods

### Isolation and biomass production of algae

**Fig. 1.**
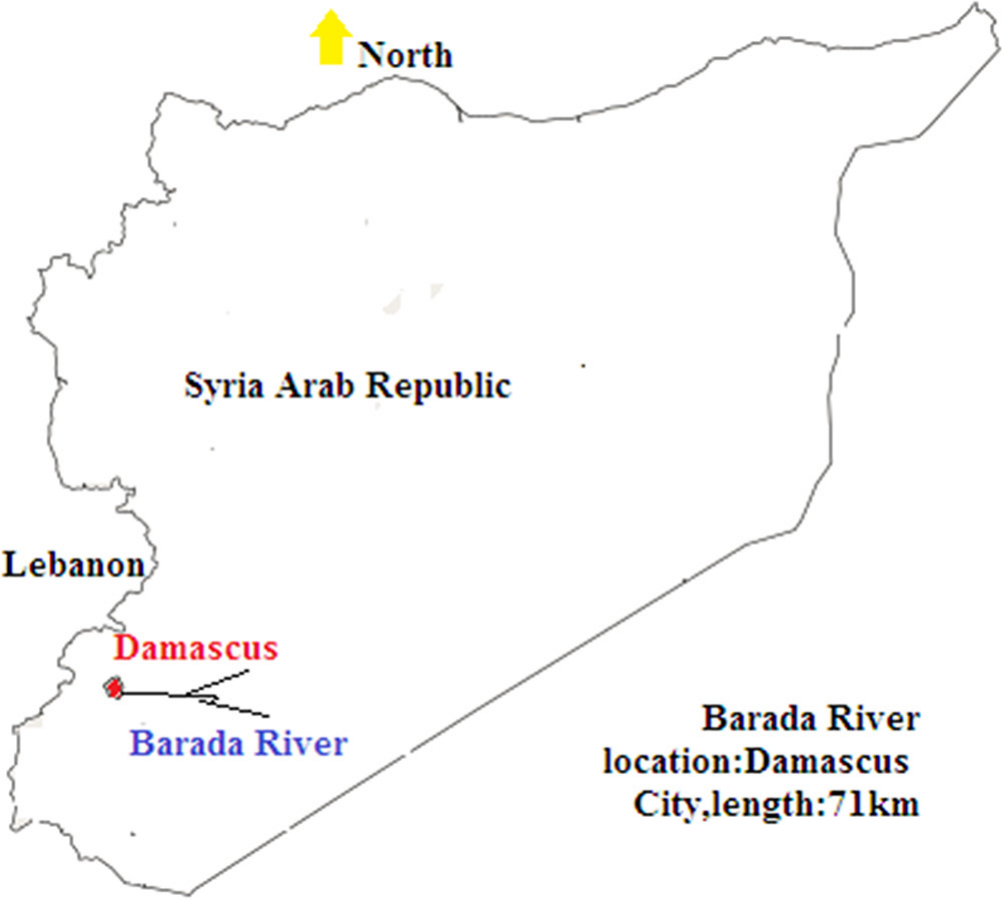
Sampling site location.

Algae samples were collected from the Barada River, Rabweh, North-West of Damascus city, Syria (Fig. 1), on 20 April 2021. Samples were kept in 5 l polyethylene bottles at 4 °C until they were shipped to the laboratory (Plant Biology Department, Faculty of Science, Damascus University, Syria), where they were washed with distilled water several times before identification; *Cladophora* cells were collected by grabber. Morphological diagnosis was performed as described by Prescott [19] by examination under a light microscope (Olympus CX41).

The identified isolates were purified by repeated cultivation in algae culture broth (Fluka medium; macronutrients NaNO_3_, KH_2_PO_4_, alkaline EDTA, acidified iron solution, boron, trace metals solution) as indicated by West [20]. A single-batch system was used for this purpose, where 10 g algae was inoculated into 100 ml medium in a 250 ml conical flask, and incubated for 14 days at 23 °C and pH 8. Cultures were aerated by aerating pumps, and illuminated by fluorescent tubes with an intensity of 1800 lux, and a light/dark cycle of 16/8 h. Algae were transferred for outdoor cultivation for biomass production during the summer using open ponds with control of the nitrogen-to-phosphorus ratio [21], as shown in Table 1; a DR 2800 Lange spectrophotometer was used to estimate these elements in the water.

**Table 1.** Treatments of outdoor cultivation for biomass production

Treatment	KH_2_PO_4_ (mg l^−1^)	Yeast extract (mg l^−1^)
1	0.4	0.4
2	0.4	0.8
3	0.4	1.2
4	0.8	0.4
5	0.8	0.8
6	0.8	1.2
7	1.2	0.4
8	1.2	0.8
9	1.2	1.2
Control (distilled water)	0	0

Algal growth rate was estimated by the method of Stein [22] based on measuring the optical density by spectrophotometer (UV/vis spectrophotometer model Optizen 2120 UV plus) at a wavelength of 650 nm. The algae biomass was isolated from the culture medium by centrifugation method at a speed of 3000 r.p.m. for 30 min, for the purpose of disassembling the strands into cells, sediment was discarded and the supernatant liquid was taken for measurement. Culture medium was used as the control solution, and the growth rate was calculated and expressed as the specific growth rate. Generation time was calculated according to the two equations developed by Huang and Wang [23]:

$$K=\frac{{log}_{ODT}-{log}_{OD0}}{T}X3.322$$

$$G=\frac{0.301}{K}$$

K is the specific growth rate (cells h^−1^); ODT, optical density at the end of the experiment; OD0, optical density at the start of the experiment; T, time of the experiment; G, generation time (h).

Biomass was harvested at the stationary phase (where optical density was constant for a period of time then declined).

### Polysaccharide extraction

Polysaccharides were extracted using the UAE technique as described by Esmaeili *et al*. [24], followed by sequential extraction according to the method of Song *et al*. [12] with some modifications. Briefly, algae dried powder was mixed with distilled water in the ratio of 30:1 (ml:g), and the mixture was put in an ultrasonic bath (Ultrasonic cleaner model ps-60ar, 360 W, 40 kHz; JeKen) for 120 min at 60 °C. Algae biomass was separated from the extract by centrifugation at 5000 r.p.m. for 10 min. Polysaccharides in the supernatants were precipitated by adding pure ethanol (99.9%) at triple the volume of the extract volume. The mixture was incubated at 4 °C for 48 h, and the pellets formed were resolved using distilled water. Lipids were removed by adding chloroform and acetone mixture (3:1), at triple the volume. The residual proteins were removed by adding Sevage reagent (1:4, v/v, mixture of *n*-butanol and chloroform) and centrifugation at 4000 r.p.m. for 15 min; the precipitate was discarded and the supernatant was dialysed for 48 h using dialysis bags. Polysaccharides were precipitated by the addition of pure ethanol three times at 4 °C for 48 h, then collected using cold centrifugation at 5000 r.p.m. for 30 min at −20 °C.

### SP purification

Purification of crude polysaccharides from *C. crispata* was carried out according to the method of Pier *et al*. [25] with some modifications; where 0.5 g crude SPs was dissolved in 100 ml phosphate-buffered saline (PBS; 6.8 g sodium chloride, 0.43 g potassium dihydrogen phosphate, 1.48 g disodium hydrogen phosphate dissolved in 1 l distilled water, pH 7.2). A total of 3 ml SPs and PBS was loaded into a gel filtration column [Sephadex G-100 gel (Sigma); column size 2.6×20 cm], and eluted with PBS at a flow rate of 1 ml min^−1^. The eluted fractions were collected in a volume of 700 µl. Polysaccharides were precipitated by the addition of pure ethanol three times and incubation at 4°C for 48 h. SP solution was then concentrated via rotary evaporation at 60 °C and 100 r.p.m. The SPs were crystallized by incubation at 50 °C with shaking at 150 r.p.m. for 12 h by using a shaking incubator (JSR model JSSI-100) until reaching the water content of 10 %.

### Fourier transform infrared spectroscopy (FT-IR)

FT-IR was used to recognize the functional groups in purified SPs. Each fraction (2 mg) from polysaccharide was dried in an oven at 40 °C for 12 h, and then mixed with potassium bromide (KBr) powder. FT-IR spectra were measured in the frequency range of 400 to 4000 cm^−1^ using a FT-IR-106 4200 type A-C077661018 instrument [12].

### Determination of SP components

The phenol/sulfuric acid method was used to determine the carbohydrate content of each fraction of SPs [26]. The sulfate content in SPs was calculated by the barium chloride gelatine assay [27]. Protein content was determined according to the Lowry method [28].

### Monosaccharide analysis

Carbohydrate analysis requires prior hydrolysis by sulfuric acid, briefly: 30 mg from each sample (crude SPs, F1 and F2) was hydrolysed with 300 µl of 72 % sulfuric acid solution for 30 min at 30 °C, and the mixture then was diluted by the addition of 8.4 ml distilled water and incubated at 121 °C for 1 h. The hydrolysed SPs were neutralized by calcium carbonate as described by Cui *et al*. [29].

The monosaccharides were selected by high performance liquid chromatography (HPLC) according to the assay of Xu *et al*. [30], with some modifications; the temperatures of the column and refractive index detector (RID) were set at 30 °C, the mobile phase was composed of acetonitrile and deionized water (85:15, v/v), flow rate was 1.0 ml min^−1^ and the injection volume was 20 µl. A standard solution (Sigma-Aldrich) was injected into the chromatography equipment at the concentration range of 0.5–50 mg in 1 ml deionized water. All samples were diluted with deionized water and filtered through 0.45 µm nylon filters prior to HPLC analysis. The experiments were repeated three times. The column was washed with the mobile phase at the end of each experiment period for more than 20 min.

### Antibacterial activity of SPs

Analysis of the antimicrobial activity of crude SP and its fractions was carried out against both Gram-positive (* Staphylococcus aureus* and *Bacillus anthracis *) and Gram-negative bacteria (* Enterobacter aerogenes* and *Pseudomonas aeruginosa *), which were obtained from the Microbiology and Algae Laboratory, Damascus University, where they were identified according to Bergey’s manual [31]. The strains were grown on nutrient agar for 24 h at 37 °C, a part of each bacterial inoculum was then taken into a sterile tube containing 5 ml nutrient broth and incubated for 4 h, with shaking at 100 r.p.m. at 37 °C [32].

#### Preparation of inoculum

The turbidity of bacterial suspension was adjusted to 0.5 McFarland scale (at OD_600_) using sterile physiological saline solution, equal to 1×10^8^ c.f.u. ml^−1^ using the turbidity method by McFarland assay; followed by dilution to a concentration of 10^5^ c.f.u. ml^−1^ after that. The bacterial suspension was then inoculated onto Mueller–Hinton agar (MHA) plates [33].

#### Antibacterial activity assay

Antibacterial activity was determined for each fraction. Firstly, 50 µl standardized inoculum of the bacterial suspension (10^5^ c.f.u. ml^−1^) was inoculated onto the MHA surface. Then, wells were made in the MHA medium using a drill bit no. 3, where the agar plugs were removed with a sterile needle [34]. A known concentration of each fraction from SPs (20, 30, 40, 50 mg ml^−1^) was inoculated into the wells. The positive control was ceftriaxone (at a concentration of 0.45%), while distilled water was the negative control. Then, the agar plates were incubated at 37 °C for 24 h, and the bacterial sensitivity was recorded. Inhibition measurement was carried out using a ruler, as the diameter of the zone sizes to the nearest millimetre [33].

#### *In vitro* minimum inhibitory concentration (MIC) determination

The MIC of SPs on the bacterial growth was determined by the agar dilution method. The SPs were dissolved in MHA medium, and serial dilution of SPs were prepared (concentration of SP solutions ranged from 0.5 to 50 mg ml^−1^). SP solutions were placed in Petri dishes and exposed to drying; there were also Petri dishes without SP solutions. Fresh bacterial suspension (10^5^ c.f.u. ml^−1^) was used (1 µl each microbial strain was added to each Petri dish), and placed in an incubator at 37 °C for 24 h. The experiments were performed in triplicate [35].

### Statistical analysis

All experiments were performed in triplicate, and experimental data are represented by the mean value ±sd of each sample. Statistical analyses were done by IBM spss version 20 using one- or three-way ANOVA at a level of significance of 0.01.

## Results and Discussion

### Algae species description and biomass production

**Fig. 2.**
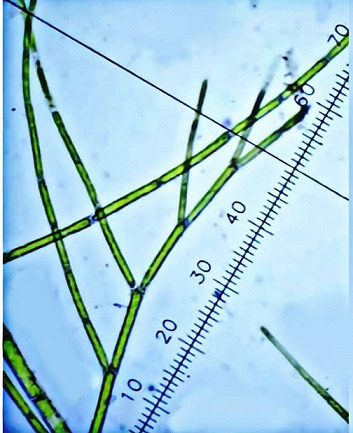
Morphology of *C. crispata* under a microscope at ×40 magnification.

Freshwater green macroalga *C. crispata* is multicellular, filamentous, truly branched, with cell patterns that are cylindrical, and the diameter of the head branches is about 50–70 μm, while ranging between 20 and 35 μm in the lateral branches, as seen in Fig. 2. The same microscopy observations have been reported by others [36]. Algae structure and a large amount of biomass production are related to habitat conditions. Depth of water, total dissolved salts, orthophosphate, nitrate chloride and Chlorophyll-a pigment content in water are key parameters to ubiquitous algae colonies [37]. In conditions such as a water temperature at 26 °C, weather temperature at 35 °C, an illumination duration of 14.30 h, the intensity of illumination 790 lux and N:P ratio 3:1, the specific growth rate was 0.3 cells h^−1^ and the generation time 0.97 h. So, our results indicate that the algae biomass was exposed to a higher temperature causing stress to the algae, this led to an increase in the carbohydrate content within their biomass; as carbohydrate synthesis requires less energy, carbohydrates were synthesized before lipids in a rapid response to environmental stress [38]. The synthesis of carbohydrates was affected by nitrogen concentration. The highest carbohydrate content (75.23%) was obtained in the culture supplemented with a higher nitrogen concentration at high temperatures, and these results were consistent with similar research [39]. A culture that was P limited (low P) led to the accumulation of carbohydrate content, this is in agreement with other work [40].

### Purification of crude SP from *C. crispata*

**Fig. 3.**
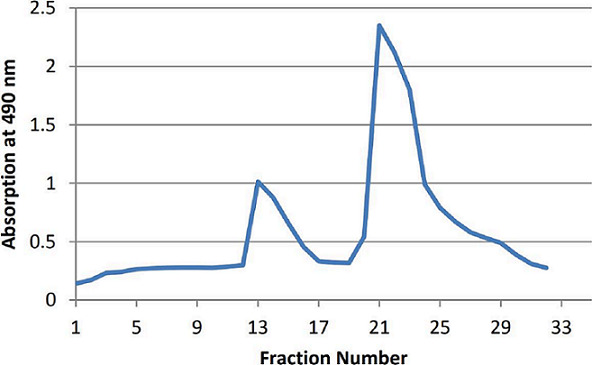
Sephadex G-100 chromatogram elution profile of crude SPs of *C. crispata*.

A sequential extraction method was used after sonication to extract a maximum amount of SPs. The SPs yield was 7.14 %, which consisted of 74.12 % carbohydrate and 9.08 % sulfate group. Purification of SPs from *C. crispat*a gave purified SPs that were well characterized (high carbohydrate and low protein contents), crude SPs were purified by gel chromatography, and fractions of SPs were selected based on the total carbohydrate elution profile (Fig. 3). The crude SP extract gave two fractions, this result is in agreement with other studies [12, 25]: F_1_ (fraction numbers 10–17) and F_2_ (fraction numbers 22–27).

### FT-IR spectra

**Fig. 4.**
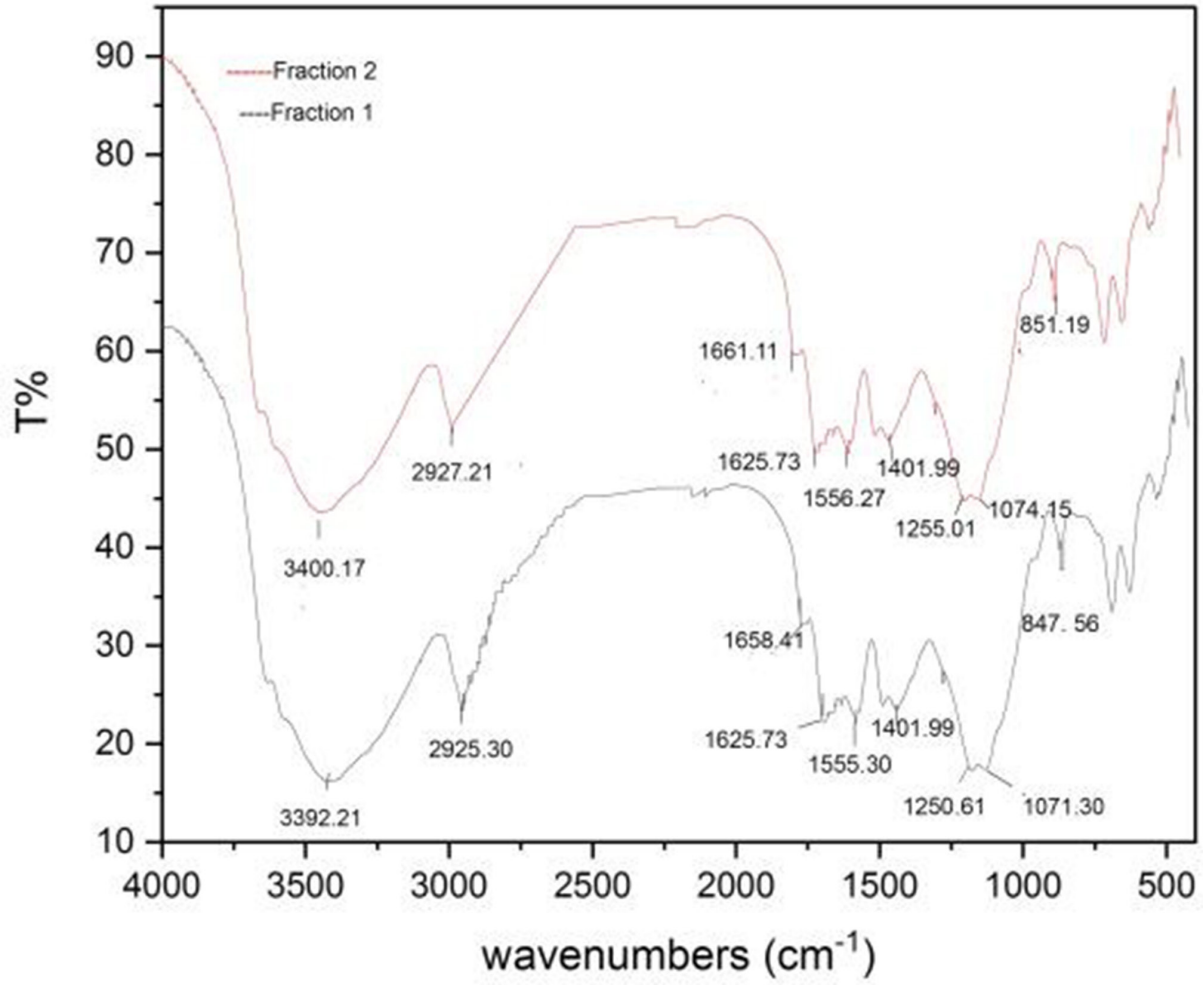
FT-IR spectra of fraction 1 and fraction 2 of purified SPs extracts from *C. crispata*.

The infrared spectra of each of the two fractions obtained from the purification of crude polysaccharides are shown in Fig. 4; both F_1_ and F_2_ have the same spectra, with little difference in the wave numbers. We noted the peak of the O–H stretches at 3397.21 and 3404.13 cm^−1^, peaks of the C–H stretching vibration are found at 2925.30 and 2926.44 cm^−1^, this is including CH, CH_2_ and CH_3_ stretching. The absorption peaks of amide I were found at 1653.66 and 1663.30 cm^−1^, and 1555.30 and 1556.27 cm^−1^ peaks were indicated to be amide II. The C=O (carboxylate groups) asymmetric stretching vibrations were observed at 1625.73 cm^−1^, it indicates the presence of uronic acids. The absorption peaks at around 1401.99 cm^−1^ refer to C–H bends, and the peaks at 1250.61 and 1252.53 cm^−1^ were due to S=O stretching vibration; also, peaks at 1077.04 and 1074.15 cm^−1^ were due to C-O. The C-O-S indicated the majority of sulfate groups, 847. 56 cm^−1^, and peaks at about 590–900 cm^−1^ refer to pyranose [12, 15, 41, 42].

### Chemical contents of purified SPs

The chemistry of the contents of crude extract and fractions F_1_ and F_2_ is shown in Table 2; we noted the carbohydrate content was associated with protein content (*r*=0.73; *R* ^2^=0.54) but not for the sulfate (*r*=−0.39; *R* ^2^=0.15) and uronic acids (*r*=−0.99; *R* ^2^=0.99). Carbohydrate, protein, sulfate groups and uronic acids contents for crude extract and fractions are not the same. A protein is contamination of cell wall SPs, because protein is a part of the structure of cell walls and was closely associated with polysaccharides. All SP extracts were contaminated with protein because of the ester sulfate moieties, which can form strong anions and attract positively charged proteins [43], after purification, the high sulfate groups are tightly linked to the SP chains [44].

**Table 2.** Chemistry of the contents of the crude extract and purified fractions of SPs (based on moisture content 10%) A, B, C, the same letters in the same column indicate no significant differences at 1%.

Component	Carbohydrate (%)	Protein (%)	Sulfate (%)	Uronic acids (%)*
**Crude SPs**	74.12±0.003 A	4.02±0.0019 A	9.08±0.002 C	2.87±0.006 C
**F_1_**	59.07±0.002 C	2.52±0.002 B	10.04±0.002 B	18.46±0.003 A
**F_2_**	65.36±0.002 B	1.83±0.002 C	12.17±0.002 A	10.62±0.003 B

*Percentage of uronic acids=100-(carbohydrate+sulfate+protein+moisture content).

### Monosaccharide composition

**Fig. 5.**
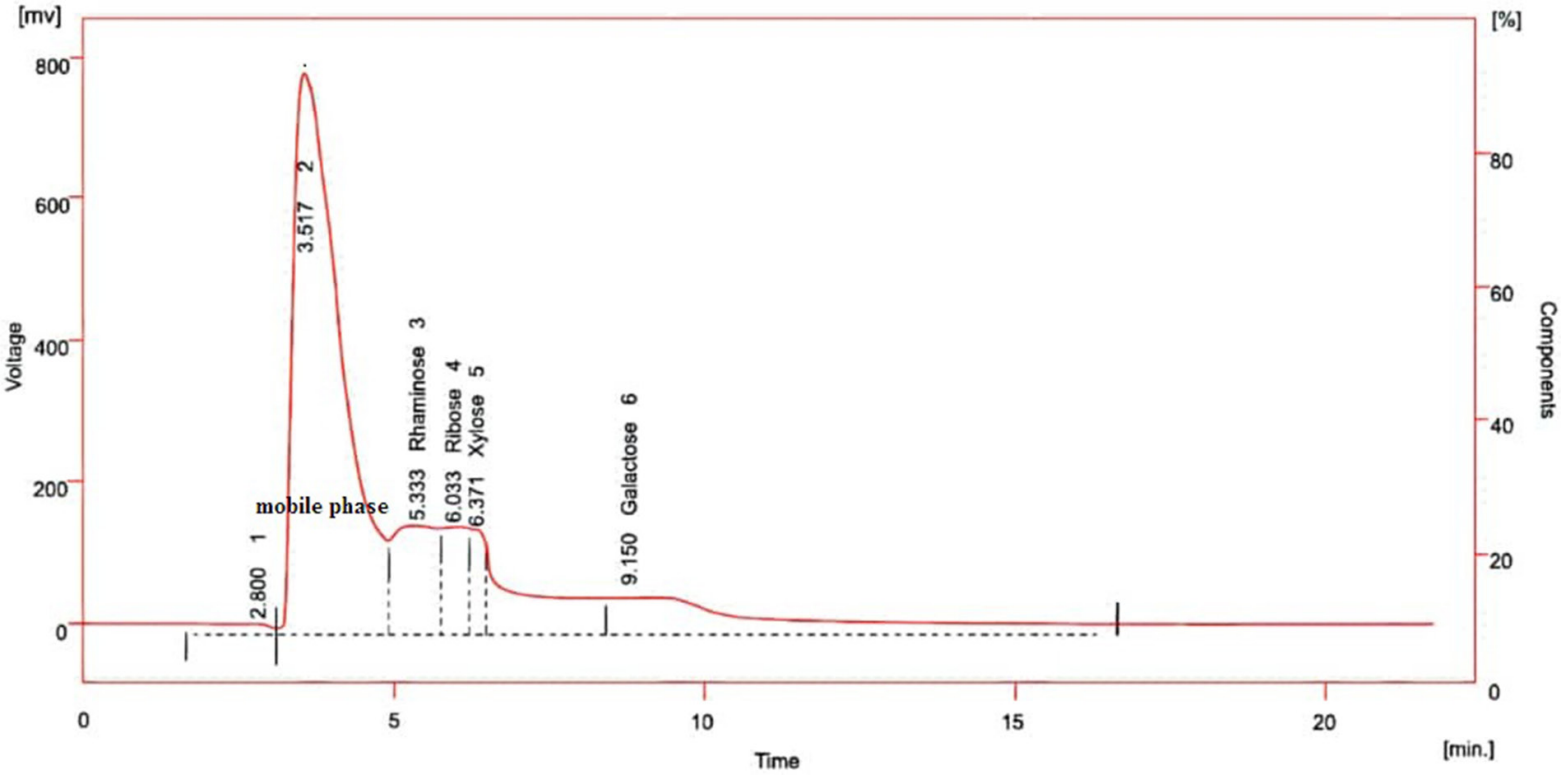
HPLC analysis of the monosaccharide composition in crude extract and fractions of SPs extracted from *C. crispata*.

The monosaccharides in the crude extract and fractions of SPs extracted from *C. crispata* were: rhamnose, galactose, xylose and ribose, based on the HPLC analysis (Fig. 5); this result is in agreement with other research [36]. The polysaccharide from green algae is ulvan. Ulvan is a heteropolysaccharide; and the composition is rhamnose, xylose, sulfate groups and uronic acids, such as iduronic acid or glucuronic acid. The composition of ulvan depends on the processing procedures of the biomass, algae species and eco-physiology. Ulvan has three types based on linked rhamnose to uronic acids; nuclear magnetic resonance (NMR) spectroscopy has been used for analysis, and other monosaccharides were reported (e.g. galactose, glucose, arabinose and mannose). This structure is common in marine and freshwater algae [9, 43, 45].

### Antibacterial activities

**Fig. 6.**
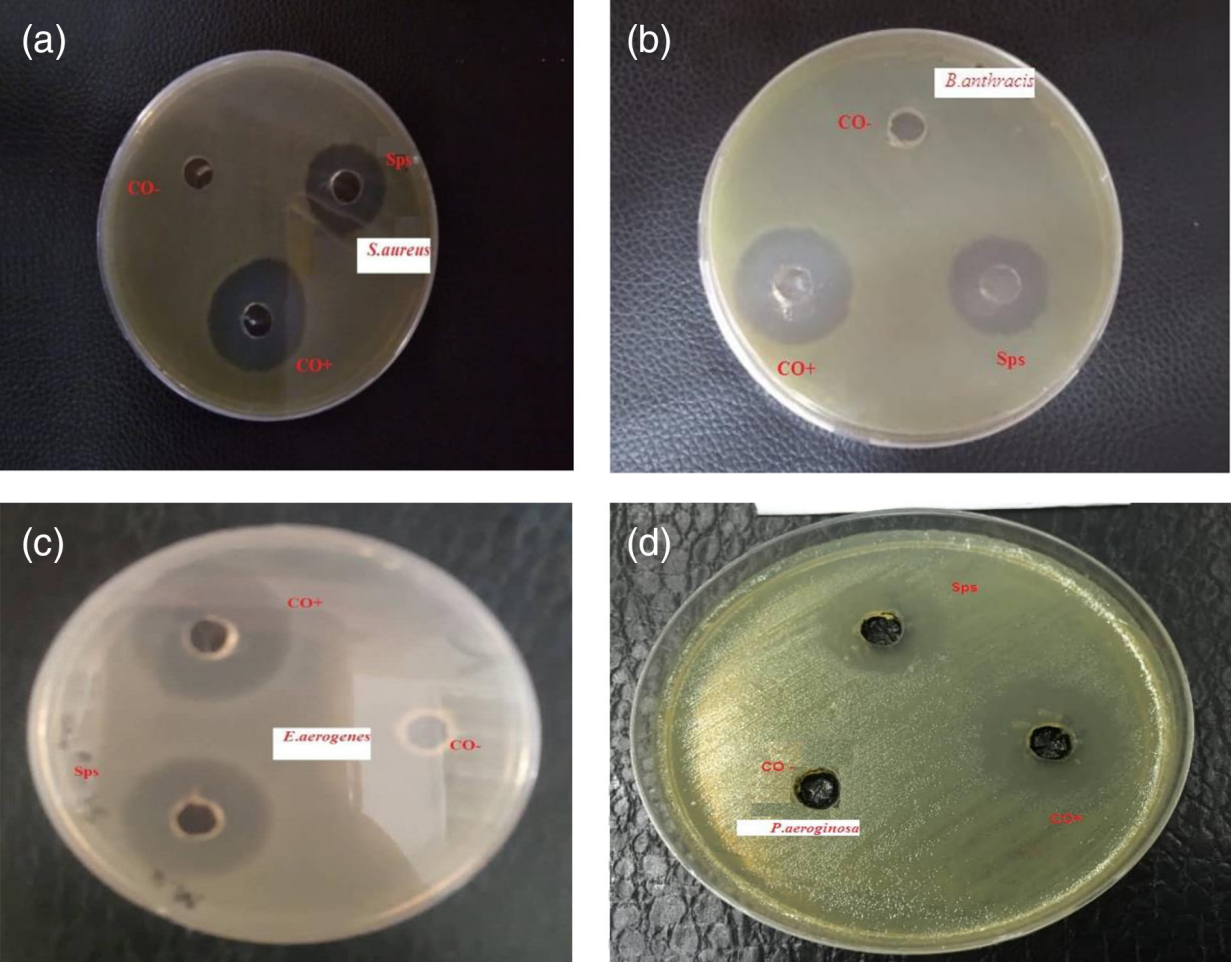
Effect of SPs against bacteria. a) *S. aureus *, b) *B. anthracis *, c) *E. aerogenes *, d) *P. aeroginosa*

The block randomization method was used in this research, not only Gram-positive but also Gram-negative bacteria strains were inhibited by purified SPs, and the antibacterial activity (inhibition zone) of the purified fractions of SPs increased with their increasing concentration, as shown in Table 3. *S. aureus* was the most sensitive to this SP, with an inhibition zone ranging from 7 to 27 mm. These results were similar to those in other reports [33]. The positive control had an effect in all bacterial strains, but not the negative control, as shown in Fig. 6.

**Table 3.** Antibacterial activity of the purified fractions of SPs from *C. crispata* A, B, C, D, E, F, G, H, the same alphabetical capital letters in the same column indicate no significant differences at 1%.

Fraction	Concn (mg ml^−1^)	Inhibition zone (mm)			
		Strains			
		* Staphylococcus aureus *	* Bacillus anthracis *	* Enterobacter aerogenes *	* Pseudomonas aeruginosa *
F_1_	20	7.0±1^ a^ F	6.6±1.1^a^ G	0^b^ G	0^b^ F
F_2_	20	8.0±0.5^a^ F	7±1^b^ G	0^c^ G	0^c^ F
F_1_	30	11.0±2^a^ E	9±2^b^ F	7.3±0.5^c^ F	0^d^ F
F_2_	30	15.0±1^a^ D	11±1^b^ E	9±1^c^ E	0^d^ F
F_1_	40	19.3±0.5^a^ C	17±1^a^ D	11±1^c^ D	14±1^c^ D
F_2_	40	27.0±1^a^ A	25.3±0.5^b^ B	14±1^d^ C	21±1^c^ A
F_1_	50	21.0±2^a^ B	19±1^b^ C	17±2^c^ B	16±1^c^ C
F_2_	50	27.0±1^a^ A	27±1^a^ A	19±1^b^ A	19.3±0.5^b^ B
Positive control	0.05	22.0±1^a^ B	18±1^c^ CD	19±3^b^ A	11±1^d^ E
Negative control	–	0^a^ G	0^a^ H	0^a^ G	0^a^ F

The antibacterial activity of algae extracts against *Enterobacter* sp. was classified as resistant or active. The purified ulvan from the green alga *Ulva reticulata* also had an antibacterial activity with an inhibition zone diameter of about 20 mm [45]. *S. aureus* and *B. anthracis* bacteria are more sensitive to antibacterial agents than *E. aerogenes* and *P. aeruginosa *, as a result of having the additional protection afforded by an outer membrane, such as lipopolysaccharide and phospholipids [46]. According to other studies, the ester sulfate of polysaccharides is related to their biological activity, such as antibacterial activities and preventing preformed biofilms [47, 48]. *S. aureus* had the lowest MIC (6 mg l^−1^) when we used F_2_; while *P. aeruginosa* had the highest MIC (32 mg ml^−1^) when we used F_1_ (Table 4). Our results suggested that the SPs might be able to activate intestinal epithelial cells to produce cell-mediated immune response cytokines that initiate and amplify protective immune responses of the host [33].

**Table 4.** MIC (mg ml^−1^) for purified SPs against *S. aureus *, *B. anthracis *, *E. aerogenes* and *P. aeruginosa
*

Bacterial strain	MIC (mg ml^−1^)	
	Fraction F1	Fraction F2
* S. aureus *	10	6
* B. anthracis *	18	13
* E. aerogenes *	28	25
* P. aeruginosa *	32	30

### Conclusions

*C. crispata* is simple to classify because it has regular branches, and its growth in the outdoor ponds systems is an important feature for this species, because of the cheap equipment required for this purpose. *Cladophora* biomass is an unlimited, easily cultivated low-cost and valuable resource for different applications. Ultrasonic waves were considered a useful method for extracting SPs from *C. crispata*, with the following conditions: ratio of 30:1 ml g^−1^, 60 °C, 120 min.

SPs from *C. crispata* are heteropolysaccharides, both F_1_ and F_2_ from purified SPs have the same monosaccharides, consisting of carbohydrate, protein, sulfate groups and uronic acids based on FT-IR analysis. It could be interesting to better purify the SP fractions by using ion-exchange resins to eliminate the presence of proteins. Purified SPs exhibit antibacterial activity because of their viscosity, water-solubility, hydroxyl and sulfate group. Mechanisms of antibacterial action for SPs are due to glycoprotein receptors present on the cell surface of polysaccharides that bind with compounds in the bacterial cell wall, cytoplasmic membrane and DNA, and cause increased permeability of the cytoplasmic membrane, protein leakage and binding of bacterial DNA. The antibacterial activity of SPs suggested possible use within the food industry, animal diets and pharmaceutical industries. We advise investigation of the antioxidant properties for SPs as further research, because the ester sulfate in SPs can donate an electron, which can give it antioxidant properties.
